# Synergistic Enhancement of Diagnostic Imaging: Synthesis and Preliminary Safety Evaluation of Gadolinium-Doped Carbon Quantum Dots as Dual-Contrast Agent

**DOI:** 10.3390/molecules29174075

**Published:** 2024-08-28

**Authors:** Marek Wojnicki, Konrad Wojtaszek, Tomasz Tokarski, Edit Csapó, Joanna Knutelska, Marek Bednarski, Alicja Skórkowska, Lucyna Pomierny-Chamioło, Magdalena Kotańska

**Affiliations:** 1 Faculty of Non-Ferrous Metals, AGH University of Science and Technology, Mickiewicza Ave., 30-059 Krakow, Poland; marekw@agh.edu.pl (M.W.); kwojtasz@agh.edu.pl (K.W.); 2 Academic Centre for Materials and Nanotechnology, AGH University of Science and Technology, al. A. Mickiewicza 30, 30-059 Krakow, Poland; tokarski@agh.edu.pl; 3 MTA-SZTE Lendület “Momentum” Noble Metal Nanostructures Research Group, University of Szeged, Rerrich B. sqr. 1, H-6720 Szeged, Hungary; juhaszne.csapo.edit@med.u-szeged.hu; 4 Interdisciplinary Excellence Center, Department of Physical Chemistry and Materials Science, University of Szeged, Rerrich B. sqr. 1, H-6720 Szeged, Hungary; 5 Department of Pharmacological Screening, Jagiellonian University Medical College, Medyczna 9, 30-688 Krakow, Poland; joanna.1.knutelska@uj.edu.pl (J.K.); marek.bednarski@uj.edu.pl (M.B.); 6 Imaging Laboratory, Center for the Development of Therapies for Civilization and Age-Related Diseases, Jagiellonian University Medical College, Medyczna 9, 30-688 Krakow, Poland; alicja.skorkowska@uj.edu.pl; 7 Department of Toxicology, Jagiellonian University Medical College, 30-688 Krakow, Poland; lucyna.pomierny-chamiolo@uj.edu.pl

**Keywords:** gadolinium-doped carbon quantum dots, carbon quantum dots, *Danio rerio*, zebrafish, toxicology, gadobutrol, dual-contrast agent, fluorescent properties, magnetic properties

## Abstract

The present study explores the synthesis and bio-safety evaluation of gadolinium-doped carbon quantum dots (GCQDs) as a potential dual-contrast agent for diagnostic imaging. GCQDs exhibit both fluorescent and magnetic properties, making them suitable for UV–Vis and magnetic resonance imaging (MRI). The synthesis of GCQDs was achieved via hydrothermal treatment, incorporating gadolinium into the carbon quantum dot matrix. The magnetic properties of GCQDs were analyzed, showing significantly enhanced values compared to gadobutrol, a common MRI contrast agent. However, synthesis constraints limit the gadolinium content achievable in nanodots. To assess the safety of GCQDs, their effects on the embryonic development of zebrafish (*Danio rerio*) were examined. Various concentrations of GCQDs were tested, observing mortality rates, hatchability, malformations, heartbeats, spontaneous movement, and GCQDs uptake. Dialysis studies indicated that gadolinium ions are incorporated into the internal structure of the carbon nanodots. Zebrafish toxicity tests revealed that while survival rates were comparable to control groups, hatchability decreased significantly with higher gadolinium concentrations in GCQDs. Fluorescence microscopy showed no statistical differences in the fluorescence intensity between groups. These findings suggest that GCQDs could serve as an effective dual-contrast agent, combining the optical imaging capabilities of CQDs with the enhanced MRI contrast provided by gadolinium. This study underscores the need for further research on the synthesis methods and biological interactions of GCQDs to ensure their safety and efficacy in medical applications.

## 1. Introduction

Carbon quantum dots (CQDs) were serendipitously discovered in 2004 during the purification of single-walled carbon nanotubes by Xu et al. [1]. These nanomaterials have since garnered significant attention due to their unique properties and potential applications in various fields, including bioimaging, sensing, and drug delivery [2]. CQDs are typically less than 10 nanometers in size [3] and exhibit excellent water solubility [4], high biocompatibility [5], and low toxicity [6,7,8], making them suitable for biomedical applications [9]. One of the most remarkable properties of CQDs is their tunable photoluminescence [10]. The emission color of CQDs is highly dependent on their size, with smaller dots generally emitting shorter wavelengths (blue light) [11] and larger dots emitting longer wavelengths (red light) [12]. This size-dependent emission is attributed to the quantum confinement effect [13]. Additionally, the emission color can be influenced by the surface functional groups present on CQDs [14]. Functional groups such as carboxyl, hydroxyl, and amine can modify the electronic structure of CQDs, leading to variations in their optical properties [10].

Numerous studies have demonstrated that the physical and chemical properties of CQDs are influenced by the choice of precursor materials and synthesis methods [15]. Common synthesis methods include hydrothermal treatment [15], microwave-assisted synthesis [16], and laser abrasion [17], each offering different advantages and leading to CQDs with distinct properties. In numerous studies, the synthesis of CQDs is categorized into green [18] and gray synthesis [19]. Green synthesis refers to the production of CQDs from biomaterials such as milk [9,20], bio-waste [21], and other organic matter [22]. This method is environmentally friendly and leverages natural resources to create functional nanomaterials. Gray synthesis, on the other hand, involves the use of naturally abundant compounds such as sugars [23], vitamin C [16,24], or proteins [9] to produce CQDs. This approach assumes that using non-toxic precursors reduces the risk of generating toxic CQDs. This assumption is valid because post-synthesis solutions often contain residual unreacted precursors. Therefore, selecting non-toxic precursors is justified to ensure the safety of the resulting CQDs. By employing non-toxic materials in the synthesis process, both green and gray synthesis methods aim to produce CQDs that are safer for biological and environmental applications. The reduced toxicity of the starting materials minimizes the risk of harmful byproducts, making these methods particularly appealing for biomedical and environmental uses.

CQDs can be synthesized using both bottom-up and top-down approaches [25]. In each method, a carbon source is necessary, typically derived from organic compounds or graphite. Bottom-up methods involve constructing carbon quantum dots (CQDs) from smaller molecular precursors. This method starts with simple organic compounds that are assembled into more complex nanostructures [26]. The process typically involves chemical reactions such as hydrothermal or solvothermal synthesis, where these organic molecules undergo dehydration, polymerization, and carbonization to form CQDs. The surface properties, including functional groups present on the CQDs, can be customized. These functional groups can enhance the solubility, stability, and biocompatibility of CQDs. Due to the precise control over size and surface properties, bottom-up methods allow for the customization of CQDs’ optical (such as fluorescence) and electronic properties. This makes them suitable for a variety of applications, including bioimaging, sensors, and optoelectronic devices.

In contrast, top-down methods involve breaking down larger carbon structures into nanoscale CQDs [25]. This approach typically starts with bulk carbon materials like graphite or carbon nanotubes. Techniques used in top-down methods include chemical oxidation, laser ablation, and electrochemical exfoliation. These processes reduce the size of the bulk materials to nanoscale particles. These methods can produce high-purity CQDs, which are essential for certain applications where contamination could affect performance.

Bottom-up synthesis methods offer significant advantages in terms of precise control over the physical and chemical properties of CQDs. By starting with small molecular precursors and building up to the desired nanostructures, researchers can tailor the size, shape, and surface characteristics of CQDs to meet specific requirements for various applications. This customization is particularly valuable in fields such as biomedical imaging, where the optical and electronic properties of CQDs play a critical role in their effectiveness and safety.

Magnetic resonance imaging (MRI) is a powerful non-invasive diagnostic tool used for imaging soft tissues with high spatial resolution. However, the inherent low sensitivity of MRI necessitates the use of contrast agents to enhance signal intensity and improve image clarity. Among the various contrast agents available, gadolinium-based compounds are most often used. Gadolinium (Gd) is preferred due to its strong paramagnetic properties, which significantly enhance the relaxation rates of nearby water protons, thereby increasing the contrast of MRI images. Furthermore, Gd-based agents are relatively non-toxic when chelated, preventing free Gd ions from causing adverse effects. It is important to note that many different metal ions exhibit strong magnetic properties; however, the key consideration in medical applications is safety. This is why gadolinium has become the primary metal used for signal enhancement in MRI.

Gd is particularly well-suited for use in MRI contrast agents due to its excellent paramagnetic properties, which significantly enhance the relaxation rates of water protons in tissues, thereby improving the clarity and contrast of MRI images [27]. Unlike other metals that might also have strong magnetic properties, gadolinium, when used in a chelated form, has a relatively low toxicity profile. Chelation prevents free gadolinium ions from causing toxicity, as free gadolinium can be harmful to the body.

The combination of strong magnetic properties and safety has made gadolinium the metal of choice for MRI contrast agents. Its ability to provide high-quality imaging while minimizing the risk to patients underscores its critical role in medical diagnostics. This balance of efficacy and safety is crucial in clinical settings, where the health and safety of patients are of paramount importance. Thanks to its low toxicity, manganese-based MRI contrast agents have also been tested [28]. Manganese—although a trace element essential for the proper functioning of the body—in high concentrations, exhibits toxic properties and can lead to serious health disorders. In addition, MRI contrast agents without gadolinium, known as gadolinium-free or non-gadolinium contrast agents, have several disadvantages. Firstly, they often have lower sensitivity and specificity compared to gadolinium-based agents, which can result in less clear or detailed images [29]. Secondly, alternative agents may have limited availability and higher costs, making them less accessible for routine clinical use. Additionally, some non-gadolinium agents might still pose safety concerns, such as nephrotoxicity or allergic reactions, although they are generally considered safer for patients with renal impairment. Overall, while gadolinium-free contrast agents offer some benefits, their limitations can impact diagnostic accuracy and clinical decision-making.

The synergistic integration of CQDs and Gd presents a promising approach to develop dual-contrast agents that combine the optical imaging capabilities of CQDs with the enhanced MRI contrast provided by Gd. This approach aims to leverage the unique properties of CQDs for fluorescence imaging while utilizing the magnetic properties of Gd for MRI, potentially providing a comprehensive diagnostic tool that can offer both high-resolution optical and magnetic images. However, it is important to note that the properties of composite materials cannot simply be assumed to be additive. This means that if a material is both magnetic and fluorescent, combining these materials does not necessarily result in a product that retains both properties. This is why our research is both crucial and pioneering. Among the rare earth elements (REE), gadolinium exhibits one of the highest magnetic moments, primarily due to its unique electronic structure. This high magnetic moment is a key factor in its effectiveness as a contrast agent in MRI. Holm (III) has indeed been tested as a potential MRI contrast agent. Research has shown that holmium-based nanoparticles can serve as effective T2-weighted MRI contrast agents, particularly useful in ultra-high-field (UHF) MRI due to their short electron relaxation times. These nanoparticles have demonstrated potential for dual-modal imaging, combining MRI with other imaging techniques like CT, enhancing diagnostic capabilities for conditions such as cancer. However, further studies are necessary to fully establish their clinical efficacy and safety compared to traditional gadolinium-based agents [30].

In contrast, CQDs are known for their excellent fluorescent properties but the presence of metal ions can sometimes quench this fluorescence. Therefore, our study carefully considers these factors to ensure the successful development of a dual-contrast agent.

In this study, we synthesize Gd-doped CQDs (GCQDs) and evaluate their safety and efficacy as dual-contrast agents. By investigating the interaction between the optical and magnetic properties of these nanomaterials, we aim to develop a novel diagnostic agent that could significantly improve the accuracy and effectiveness of medical imaging techniques. We aim to develop GCQDs that maintain strong fluorescence for optical imaging and high magnetic responsiveness for MRI, ensuring their effectiveness as a dual-contrast agent. This could potentially revolutionize medical imaging by providing a more accurate and detailed diagnostic tool that combines the strengths of both fluorescence and magnetic resonance imaging. Our research involves extensive characterization of the synthesized materials to understand how the integration of Gd affects the fluorescence and magnetic properties of CQDs. We employ various techniques to analyze the structural, optical, and magnetic properties of the GCQDs. Following the synthesis and characterization, we conducted screening toxicity studies to ensure that the new dual-contrast agents are safe for biological applications.

## 2. Results

### 2.1. Magnetic Properties Determination

Measurements of the magnetic properties of selected colloids and solutions were performed. The values in Table 1 present the apparent changes in sample mass that were corrected for the magnetic susceptibility of the basic measurement, which was a cuvette with distilled water. After subtracting the water component and the quartz cuvette, the values of the apparent change in mass for carbon nanodots and carbon nanodots doped with gadolinium in the range of GCQD_1–GCQD_5 (Carbon:Gadolinium atomic ratio 100:1 to 100:5) indicate that the observed Δm values are primarily due to the presence of gadolinium ions. It is significant that the magnetic susceptibility of the samples comes mainly from gadolinium ions, which can be seen by comparing the obtained values of the apparent change in mass for the GCQD_10 (Carbon:Gadolinium atomic ratio 100:10) colloid and the GdCl_3_ solution, for which the gadolinium concentration is similar. The highest value of interaction with the magnetic field was recorded for gadobutrol. The GCQD_10 nanodot solution showed the strongest magnetic properties, but compared to the value obtained for gadobutrol, this is still a relatively low value. However, it should be noted that the concentration of gadolinium in the gadobutrol solution is as much as 1 mol/dm^3^. The GCQD_10 sample shows approximately 2.5 times stronger magnetic properties than gadobutrol when normalized to the gadolinium concentration. This highlights the significant impact of gadolinium concentration on magnetic properties and illustrates the effectiveness of gadolinium-doped carbon nanodots in enhancing magnetic interactions, even though the absolute values are still lower than those of gadobutrol. Currently, there are significant challenges in synthesizing nanodots with gadolinium content as high as that found in gadobutrol, a clinically used contrast agent. Achieving similar gadolinium levels in nanodots faces technological limitations with current synthesis methods. A partial solution might involve synthesizing colloids with lower gadolinium concentrations while increasing the administered volume. However, at this stage of our research, we have focused on investigating the mechanism and toxicity of the nanodots. Nevertheless, it is a concept worth further research.

**Figure 1:**
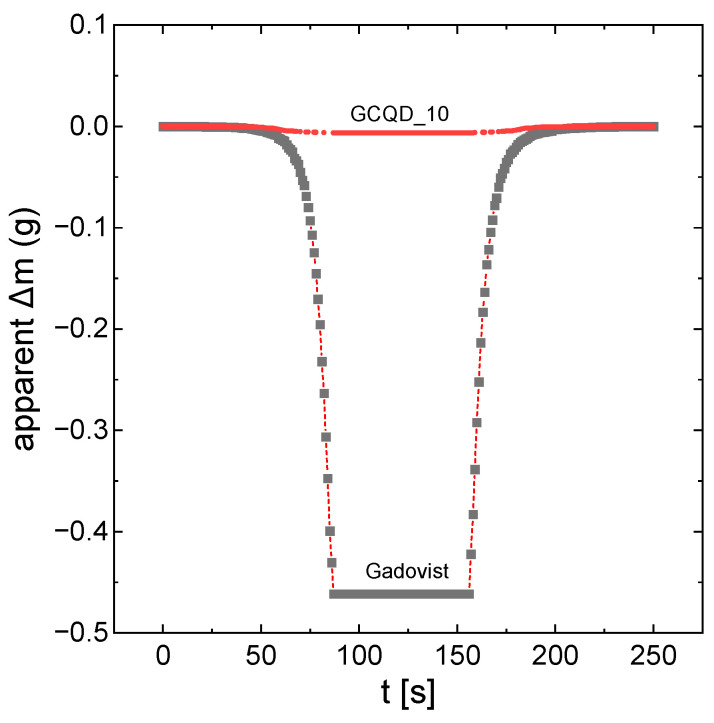
Apparent mass changes in the samples of gadobutrol and solution GCQD_10 as a function of the measurement of time.

Figure 1 shows the apparent mass changes in the samples of gadobutrol and solution GCQD_10 as a function of the measurement of time. The apparent mass changes are directly proportional to the magnetic properties of the system. As already mentioned, gadovist apparently has much stronger magnetic properties. However, after taking into account the degree of dilution, GCQD_10 interacts more strongly with the magnetic field.

### 2.2. Dialysis

The solutions after dialysis were analyzed for gadolinium content. The results presented in Table 2 show that gadolinium passes through the dialysis membrane to some extent. This means that this part of the gadolinium remained after synthesis in ionic form and did not build up in the dots but adsorbed on their surface. On the other hand, some of the gadolinium after dialysis remained in the solution with the dots in a greater amount than would result from the law of division and equalization of the concentrations of the dialyzed r-r and the water in which the dialysis was conducted. This is evidence that gadolinium is incorporated into the internal structure of carbon nanodots.

Based on the difference in gadolinium content in the sample before and after dialysis, the gadolinium loss was determined and the new atomic ratio of gadolinium to carbon in the nanodots was calculated. It can be assumed that the new atomic ratio refers strictly to gadolinium incorporated into the internal structure of the nanodots.

### 2.3. Optical Analysis of CQDs and GCQDs

Absorption spectrum analyses of CQDs and GCQDs were conducted, revealing slight differences between the spectra. The peak maximum for CQDs is positioned at 279.5 nm, whereas for GCQDs, it is at 277.8 nm. Additionally, the GCQDs spectrum, despite having equal precursor content for the quantum dots, is approximately 25% more intense. It is important to highlight that the addition of gadolinium chloride to the reaction system changes the dielectric constant of the electrolyte. In microwave synthesis, this alters the penetration depth and absorption level of the microwave radiation, potentially causing the reactions to proceed differently.

**Figure 2:**
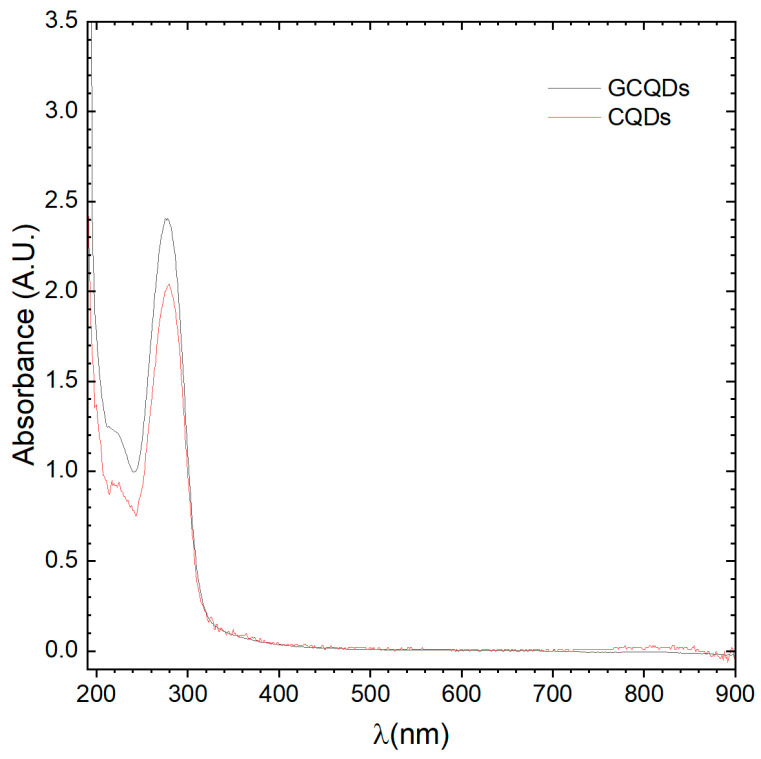
UV–Vis spectra of CQDs and GCQDs_10.

At this stage, we can already observe the impact of gadolinium doping on the optical properties; however, we cannot yet definitively determine whether gadolinium is merely associated with the surface of the CQDs or incorporated into their structure. Typically, interactions observed within this wavelength range are of the π-π* type, characteristic of CQDs (see Figure 2). The observed peak shift could indeed be a result of effective doping. These findings indicate that the presence of gadolinium influences the electronic environment of the CQDs, modifying their optical behavior, and suggesting that further investigation is required to elucidate the precise nature of this doping effect.

**Figure 3:**
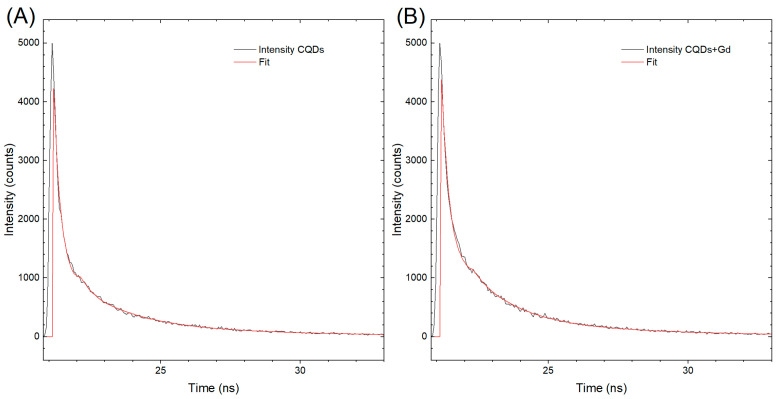
Kinetic decay curves of the excited state for (**A**) CQDs and (**B**) GCQDs_10.

The results of the excited-state lifetime measurements are presented in Figure 3, which shows two kinetic decay curves, labeled A and B, corresponding to CQDs and GCQDs_10, respectively. Although there is practically no difference in the shape of the curves, the decay curve for CQDs exhibits a faster decline. This observation suggests that the presence of gadolinium in GCQDs_10 influences the excited-state dynamics.

The half-life of the photoemission decay for CQDs is measured to be 2.38 ns. The faster decay observed in CQDs can be attributed to several factors. One possible explanation is that the incorporation of gadolinium in GCQDs_10 alters the electronic environment, potentially creating additional pathways for non-radiative relaxation, which can prolong the excited-state lifetime. Gadolinium ions might interact with the carbon quantum dots, affecting their energy levels and reducing the probability of non-radiative decay processes that typically dominate in undoped CQDs.

Additionally, gadolinium doping might introduce defects or alter the surface states of the quantum dots, which can also contribute to a slower decay of the excited state. These interactions may lead to a stabilization of the excited state, resulting in a longer emission lifetime compared to undoped CQDs.

In summary, the slight prolongation of the excited-state lifetime in GCQDs_10, as indicated by the slower decay curve compared to CQDs, is likely due to the influence of gadolinium on the electronic and surface properties of the quantum dots. This effect underscores the impact of doping on the photophysical behavior of CQDs and opens up further avenues for investigating the mechanisms underlying these observations.

**Figure 4:**
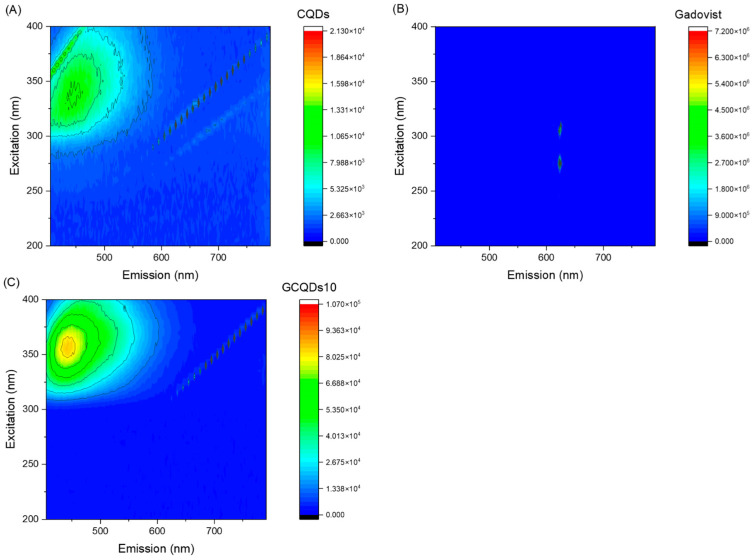
Fluorescence spectra of (**A**) CQDs, (**B**) gadovist, and (**C**) GCQDs_10.

Next, fluorescence analyses of both samples were conducted. Figure 4 presents the excitation vs. fluorescence map for gadovist, CQDs, and GCQDs_10.

The GCQDs_10 exhibit a significantly higher maximum fluorescence intensity (85,188) compared to the CQDs (11,053). This indicates that doping CQDs with gadolinium substantially enhances their fluorescent properties. The shift in the excitation wavelength from 337 nm for CQDs to 357 nm for GCQDs_10 suggests that the presence of gadolinium affects the electronic structure of the quantum dots, altering their optimal excitation wavelength. The emission wavelength for GCQDs_10 (441 nm) is slightly blue-shifted compared to that of CQDs (446 nm). This minor shift in emission wavelength further supports the conclusion that gadolinium doping modifies the electronic environment of the CQDs.

The data suggest that gadolinium doping significantly enhances the fluorescent properties of CQDs, evidenced by the increased fluorescence intensity and shifted excitation wavelength. This enhancement makes GCQDs_10 a promising dual-contrast agent for imaging applications, offering both fluorescent and magnetic properties, unlike the commercial gadovist, which does not exhibit fluorescence. Further optimization and investigation into the mechanisms of this enhancement will be crucial for developing advanced diagnostic tools.

The quantum yield of fluorescence was measured for both CQDs and gadolinium-doped CQDs, yielding values of 0.38% and 0.41%, respectively. Notably, the increase in the atomic percentage of the gadolinium dopant did not result in a significant enhancement in the quantum yield. This observation is particularly interesting given that CQDs are often considered potential sensors for heavy metal ions. Typically, the presence of heavy metal ions leads to fluorescence quenching, which is characterized by a decrease in quantum yield.

The relatively stable quantum yield in the presence of gadolinium suggests that the doping process does not introduce significant non-radiative pathways that would otherwise lead to quenching. This stability could be advantageous in applications where maintaining fluorescence efficiency is crucial, despite the presence of metal ions.

Moreover, this finding implies that GCQDs_10 retain their potential as fluorescent probes even when doped with gadolinium, without the adverse effects commonly associated with heavy metal–ion interactions. The ability of GCQDs to maintain a consistent quantum yield while incorporating gadolinium opens up further research opportunities. It would be valuable to investigate whether similar effects are observed with other dopants and to understand the underlying mechanisms that allow GCQDs_10 to preserve their fluorescence properties.

In summary, the measured quantum yields of 0.38% for CQDs and 0.41% for GCQDs_10 indicate that gadolinium doping does not significantly alter the fluorescence efficiency. This result underscores the potential of GCQDs_10 in applications requiring stable fluorescent properties in the presence of metal ions, and it challenges the common notion that heavy metal doping necessarily leads to fluorescence quenching.

### 2.4. DLS and HR-TEM Analysis

**Figure 5:**
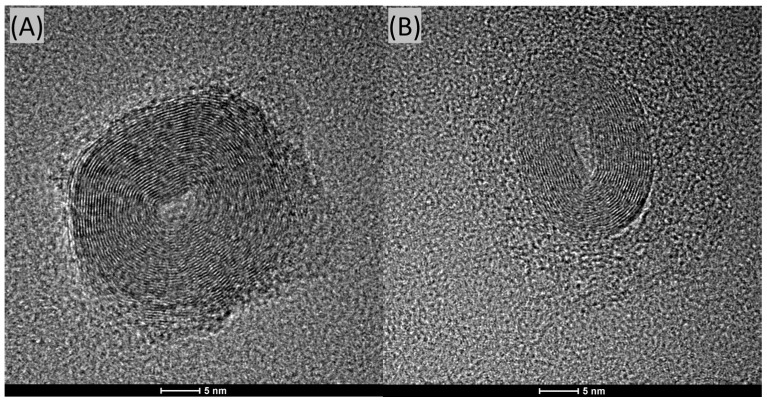
HR-TEM analysis of CQDs, where (**A**,**B**) are randomly selected detected nanoparticles.

We conducted HR-TEM analyses of the CQDs, and representative images are shown in Figure 5A,B. In both cases, it is evident that these nanoparticles possess a layered structure. This structure can be visually compared to that of an onion. These particles are relatively large, significantly exceeding the 10 nm size criterion typically used to define quantum dots.

The formation of CQDs with a layered structure may be attributed to several mechanisms during the synthesis process. One possible explanation is the nature of the carbon precursors and the conditions of the hydrothermal synthesis. During the synthesis, carbon atoms can self-assemble into graphitic layers due to strong sp2 hybridization, resulting in a layered or “onion-like” structure.

Additionally, the synthesis conditions, such as temperature, pressure, and the presence of certain catalysts or dopants (e.g., gadolinium), can influence the stacking and orientation of these graphitic layers. High temperatures and pressures can facilitate the carbonization process, leading to the formation of multiple graphitic shells around a core, akin to the layers of an onion.

Furthermore, the kinetics of the nucleation and growth phases during synthesis can also play a critical role. Rapid nucleation followed by slow growth can favor the formation of layered structures as carbon atoms continue to deposit on existing nuclei, forming concentric shells. This growth mechanism is supported by the observation of large particle sizes in the HR-TEM images, suggesting that once nucleation occurs, additional carbon layers continue to form, leading to particles that exceed the typical quantum dot size of 10 nm.

In conclusion, the HR-TEM images reveal that the CQDs possess a distinct layered structure, significantly larger than traditional quantum dots. The formation of these structures can be attributed to the self-assembly of carbon atoms into graphitic layers under specific synthesis conditions, as well as the nucleation and growth kinetics during the hydrothermal process. Further research into the precise conditions and mechanisms governing this structural formation could provide deeper insights and enable more controlled synthesis of CQDs with desired properties.

The results obtained from the Dynamic Light Scattering (DLS) method confirm the presence of particles primarily around the size of ~1.0 nm. This measurement lies at the lower detection limit of the instrument. It is also important to note that locating CQDs on a 20–30-nanometer-thick carbon grid using HR-TEM is almost impossible due to the very low contrast.

It is important to highlight that CQDs are deposited onto electron microscopy grids, which are themselves made of amorphous carbon with a thickness of approximately 20 nm; observing contrast between the CQDs and the substrate can be extremely challenging due to this factor. The amorphous nature of the carbon film and its considerable thickness relative to the size of the CQDs make it difficult to clearly distinguish between the CQDs and the underlying support.

When the particles are amorphous and significantly smaller than the thickness of the carbon film on the microscopy grid, it becomes nearly impossible to resolve them accurately using HR-TEM. This limitation can contribute to the difficulty in detecting and measuring very small CQDs, as their contrast against the substrate is minimal.

Therefore, while HR-TEM provides valuable information, the inherent challenges in distinguishing small, amorphous particles from the carbon film on the grid must be considered when interpreting the results.

### 2.5. Zebrafish Toxicity Test

**Figure 6:**
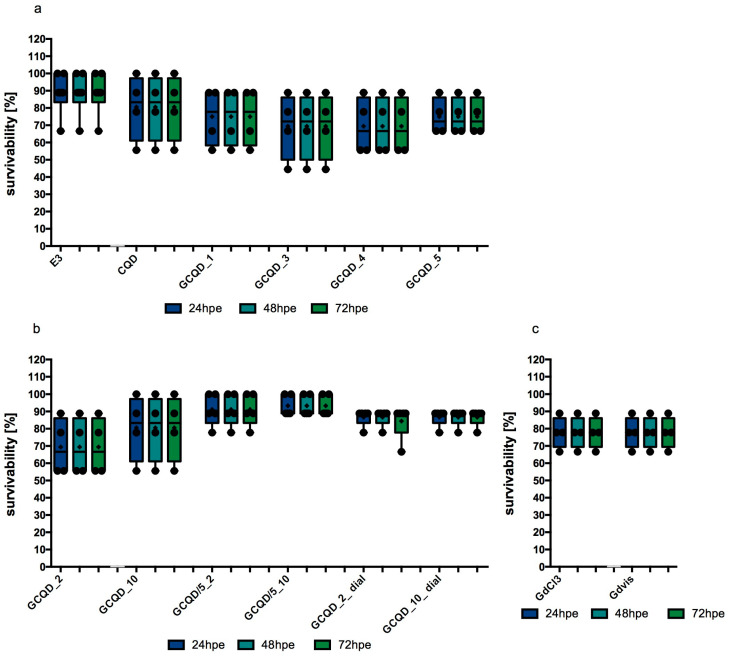
Survival of *Danio* embryos for 72 h of exposure to the tested substances: E3, CQD, GCQD_1–GCQD_5 (**a**); GCQD_2, GCQD_10, GCQD/5_2, GCQD/5_10, GCQD_2_dial, GCQD_10_dial (**b**); GdCl_3_, Gdvis (**c**). Mean (marked with “+”) ± standard deviation (SD, box), median (marked with a line), whiskers indicate minimum and maximum values, n = 4–6 (total embryos 36–54 per group); Kruskal–Wallis test. E3—buffer for embryos, CQD—quantum carbon dots, GCQD—gadolinium carbon quantum dots, GdCl_3_—gadolinium chloride, Gdvis—gadobutrol.

The median and mean survival of *Danio* embryos in the control group, staying in an appropriate embryo buffer, was 88.89%. Exposure to CQDs without gadolinium slightly decreased this survival rate, the calculated median in this group was 83.34% and the mean was 80.56%. In study groups exposed to GCQDs with different gadolinium contents (GCQD_1, GCQD_3, GCQD_4, GCQD_5), the mean survival was between 69 and75% and the median was between 66 and 78%. The results are presented in Figure 6a.

The mean survival rate of embryos exposed to GCQD_2 was just over 69% (with a median of over 66%). When embryos were exposed to a five times lower concentration of dots with the same amount of gadolinium (GCQD/5_2), the average survival rate increased to over 91% (with a median of over 88%). For embryos exposed to GCQD_2_dial, the average survival rate was around 85% (with a median of 88.89%). These findings are illustrated in Figure 6b.

When embryos were exposed to GCQD_10, the mean survival rate was around 80% (median 83.34%); however, when the embryos were exposed to a five times lower concentration of dots with the same amount of gadolinium (GCQD/5_10), the average survival rate increased to over 93% (median above 88%). Furthermore, when embryos were exposed to GCQD_10_dial, the average survival rate was approximately 85% (median 88.89%). Results are illustrated in Figure 6b.

The average and median survival calculated for embryos exposed to gadolinium chloride (in a gadolinium concentration identical to that in GCQD_10 and in the gadolinium solution used) was 77.78% (median 83.34%). Identical results were obtained for gadobutrol. The results are presented in Figure 6c.

Statistical analysis performed using the Kruskal–Wallis test showed the significance of differences at the level of *p* = 0.0149, however, Dunn’s post hoc test did not show significant differences in the multiple comparative analysis between groups.

**Figure 7:**
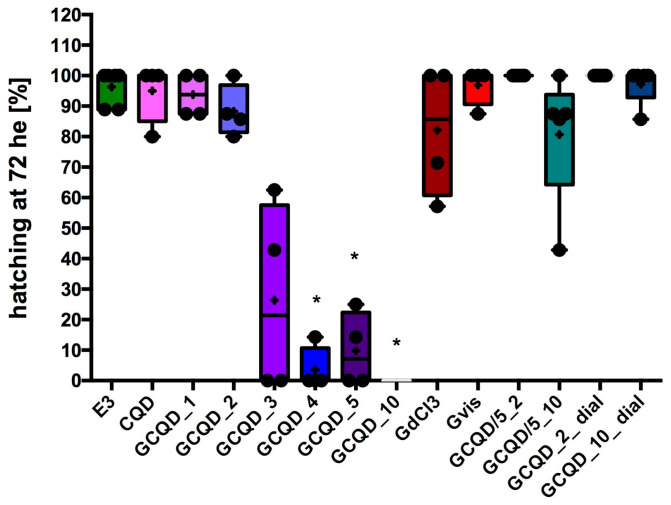
Hatchability of *Danio* embryos after 72 h of exposure to the tested substances. Mean (marked with “+”) ± standard deviation (SD, box), median (marked with a line), whiskers indicate minimum and maximum values, n = 4–6 (total embryos 36–54 per group); Kruskal–Wallis test, Dunn’s post hoc, differences vs. control (only E3) were considered at *p* < 0.05 (*). E3—buffer for embryos, CQD—quantum carbon dots, GCQD—gadolinium carbon quantum dots, GdCl_3_—gadolinium chloride, Gdvis—gadobutrol.

The hatchability of *Danio* embryos decreased in groups exposed to GCQD depending on the concentration of gadolinium used. In the groups exposed to GCQD_4, GCQD_5, or GCQD_10, hatchability was statistically significantly lower than the hatchability in the control group, and in the group exposed to GCQD_10, no embryos hatched up to 76 hpf (72 h of exposition, he). Interestingly, in the groups exposed to GCQD/5_10 and those exposed to GCQD_10_dial, the degree of hatchability was not statistically different from the hatchability determined in the control group. Not all embryos left chorions in the group exposed to gadolinium chloride. The median hatchability for this group was approximately 85%. However, the hatchability in the group exposed to gadobutrol was comparable to the hatchability determined in the control group. The results are presented in Figure 7.

**Figure 8:**
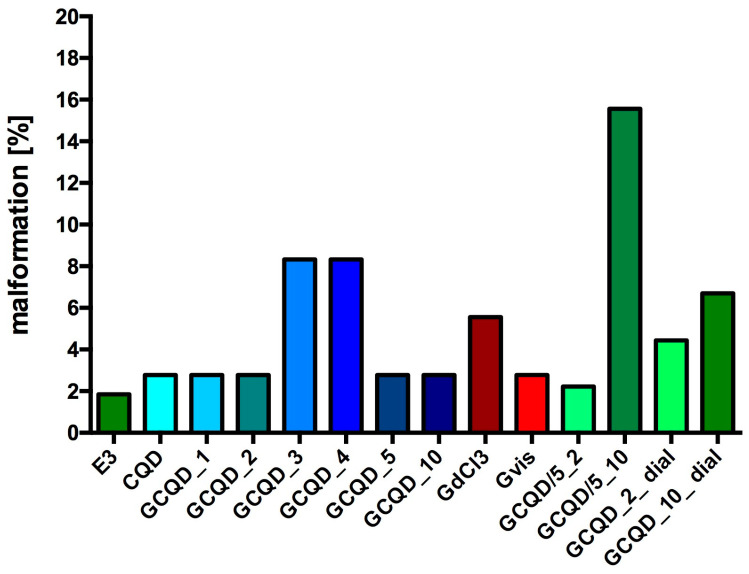
Percentage of disorders counted in fish in individual groups. E3—buffer for embryos, CQD—quantum carbon dots, GCQD—gadolinium carbon quantum dots, GdCl_3_—gadolinium chloride, Gdvis—gadobutrol; total *Danio* in each group: 36–54.

**Figure 9:**
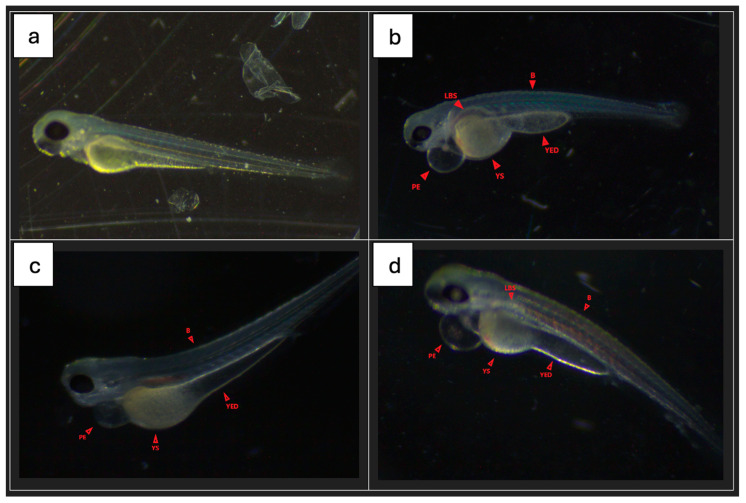
Sample photos of selected fish without and with characteristic malformations. E3 group (**a**), GCQD_3 group (**b**), GCQD_5 group (**c**,**d**). B—bent spine; YED—yolk extension; YS—yolk sac edema; PE—pericardial edema; LSB—lack of swim bladder. Zoom applied 40×.

Most fish from all groups had no signs of malformations or serious disorders. Single specimens with clearly visible changes were observed in all groups. Figure 8 shows the percentage of fish with disorders in each group (72 he), while Figure 9 shows sample photos of normal fish and those with serious disorders (pericardial edema, yolk swelling, sac edema, yolk extension deformation, lack of swim bladder, bent spine) from a light microscope. All fish without malformations had a normal heart rhythm (72 he), and their spontaneous motility (48 he and 72 he) was not disturbed.

**Figure 10:**
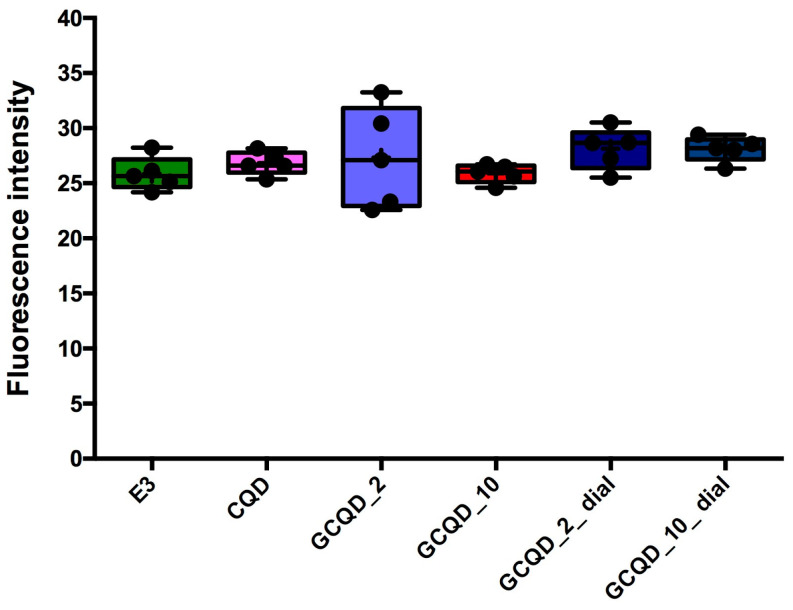
Fluorescence intensity of zebrafish larvae in blue light (4 dpf). Mean (marked with “+”) ± standard deviation (SD, box), median (marked with a line), whiskers indicate minimum and maximum values, n = 5; Kruskal–Wallis test, Dunn’s post hoc. E3—buffer for embryos, CQD—quantum carbon dots, GCQD—gadolinium carbon quantum dots, GdCl_3_—gadolinium chloride.

**Figure 11:**
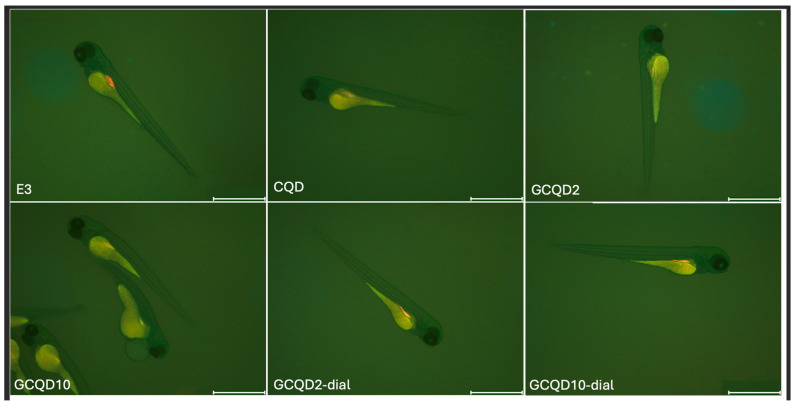
Sample photos of selected 4 dpf larval zebrafish. Visualization using a fluorescence microscope. Scale bar: 1000 μm.

Fluorescence intensity, read with a microscope and measured with a computer program, did not differ statistically significantly between groups, the Kruskal–Wallis test showed no differences in medians (*p* = 0.2853). Figure 10 shows the results of fluorescence intensity in blue light, and Figure 11 shows sample photos of selected fish.

### 2.6. Determination of Gadolinium Content in Embryos/Larvae

Spectrometric analysis of solutions after embryo mineralization after toxicity sampling revealed trace contents of gadolinium in some experimental series. The use of the comparative method in relation to the signal recorded for blank and control samples allows for determining the concentration below 0.1 ppm. The results are presented in Table 3, Table 4 and Table 5. Each sample contained 8–10 pieces of zebrafish embryos. Assuming a dry mass of one embryo of 63.2 μg [31,32], the gadolinium content was converted to the dry mass of the zebrafish embryo/larva.

## 3. Discussion

Our research shows that it was possible to synthesize GCQDs using a sucrose solution as a starting substrate, which exhibited magnetic and fluorescent properties. Combining fluorescent and magnetic properties in one marker may represent a significant advancement in the battle against civilization diseases, particularly cancer. This innovative approach may pave the way for new diagnostic and therapeutic tools. Therefore, research on the effectiveness and safety of such particles is very important.

As previously mentioned, gadobutrol exhibits weaker magnetic properties compared to GCQD_10 when considering the gadolinium concentration in both materials. This highlights the fascinating properties of gadolinium-modified quantum dots. It is also worth noting that quantum dots are significantly smaller than typical nanoparticles, meaning their diffusion rate can be comparable to that of small molecules like glucose. The diffusion rate is crucial as the contrast agent should be quickly distributed and eliminated from the body to avoid toxic effects. This is another argument supporting the use of quantum materials in medicine.

Dialysis studies of the dots clearly showed that gadolinium is incorporated into the internal structure of carbon nanodots. It is significant that at a higher initial concentration of gadolinium in the sample, the loss of gadolinium during dialysis is much lower. This suggests that at an appropriately high concentration of gadolinium, the incorporation into the nanodot structure may occur with greater efficiency. This may also result from the synthesis methodology. The ions absorb microwave radiation. A higher gadolinium concentration may translate into changes in the thermal conditions of synthesis, prior to local overheating of the precursor solution, leading to the formation of many hot spots in which the nucleation of carbon nanodots occurs, encapsulating gadolinium ions and locking them in the internal structure.

To evaluate the bio-safety of GCQDs, we studied their effects on the embryonic development of zebrafish. In line with the literature, the zebrafish is a reliable and convenient model for assessing potential nanotoxicity caused by CQDs exposure [33]. Zebrafish are used in animal-based research due to benefits such as ease of handling, low cost and time requirements, high genetic similarity with humans, and embryo transparency. These features allow real-time analysis in various areas of biology and medicine [34,35,36,37].

In this study, after zebrafish embryos were exposed to GCQDs (Carbon:Gadolinium atomic ratio: 100:1, 100:2, 100:3, 100:4, 100:5, 100:10, 20:2, 20:10, 100:2 after dialysis, 100:10 after dialysis) their mortality was not statistically significantly different than the mortality of embryo from the control group and accounted for not more than 31%. In most groups, the survival average was about 80%, which was comparable to the survival calculated after exposure to GdCl_3_ or gadobutrol. It should be noted that mortality did not increase with exposure time in any group, practically, all embryos that survived the first 24 h survived until the end of the experiment, i.e., up to 76 hpf. Then, in most groups, most of the larvae after 72 he developed physiologically, no malformations were observed in them, and the percentage of disorders was calculated at a level below 5–10. In only one group (GCQD/5_10) it was at 16%.

Interestingly, *Danio* hatchability significantly decreased in comparison to the control, depending on the concentration of gadolinium contained in the CQDs but not depending on the dots’ concentration. In the GCQD_10 group, no larva hatched on its own until 76 hpf. Compared to the group of fish that were incubated with GdCl_3_ at a concentration comparable to the highest used in CQDs, i.e., as in the GCQD_10 group, hatchability was dramatically different. However, after hatching these spontaneously unhatched larvae using tweezers, it turned out that the larvae did not have any serious developmental delays (only a slight narrowing of the yolk was observed). This shows that connecting the dots with gadolinium dramatically reduces the ability of the larvae to hatch. It is therefore likely that connecting the dots with gadolinium in large amounts changes the function or structure of the chorion.

In subsequent studies, embryos and larvae were collected after 24 h, 48 h, and 72 h to assess the gadolinium content. Interestingly, after 24 h, gadolinium was not determined in embryos exposed to GCQD_2 or in those exposed to gadobutrol. However, the gadolinium concentration in embryos exposed for 24 h to GCQD_2 and previously undergoing dialysis was similar to the gadolinium concentration determined in embryos exposed to GCQD_10 and GCQD_10 after dialysis. Additionally, the concentrations in embryos in the GCQD_10 and GCQD_10_dial groups were comparable, although dialysis resulted in a loss of approximately 15% of the gadolinium content in the solution.

Further, looking at the results after 48 h of incubation (the solutions were changed twice), the gadolinium content was determined only in groups incubated with solutions after dialysis or in which the ratio of dots to gadolinium was 20:2 and 20:10 (GCQD/5_2 and GCQD/5_10) and incubated with GdCl_3_ solution. Therefore, the difference is that gadolinium was not determined in the GCQD_10 group. Keeping in mind that although no comprehensive investigation of chorion permeability or sensitivity to chemicals has yet been reported, the chorion is suspected to be a barrier to the entry of nanoparticles into zebrafish embryos [38]. However, it is known that the chorion possesses canals, the pore size of which is approximately 0.6–0.7 μm [38], larger than the size of GCQDs. Our results therefore show that gadolinium dots pass through the chorion, but in high concentrations of dots and gadolinium, can affect the function or structure of the chorion. However, it is interesting that the GCQD_10_dial solution can pass through the chorion. Interestingly, the amount of gadolinium in samples from the GCQD/5_2 and GCQD/5_10 groups increased compared to the amount measured 24 h earlier, and the amount of gadolinium in samples from the GCQD_2_dial and GCQD_10_dial groups decreased compared to the amount measured 24 h earlier.

After 72 h of incubation with the tested solutions, the presence of gadolinium was not determined in any group of larvae. These results are consistent with the results obtained from the fluorescence microscope, which showed that after 72 h of exposure to the tested solutions, no differences in fluorescence were observed between the groups and between the fish and the background.

These studies are preliminary studies and after conducting them, there is an obvious need to examine kinetic parameters such as absorption, distribution, metabolism, and excretion. We do not yet know anything about the possibilities of effective entry of GCQDs into the embryo or larva, the period of presence of these molecules in the body, or even how GCQDs are excreted from the body of zebrafish. Studies after injecting GCQDs into the larval yolk will be necessary to obtain this knowledge.

In our research, we used immersion administration that did not ensure penetration into the body of the fish, which turned out to be a limitation of these studies. In previous studies, analogously synthesized dots (with milk used as a substrate) passed into cells [9], therefore, in these preliminary studies, we chose immersion administration, assuming that dots synthesized on the basis of sucrose doped with gadolinium would pass into embryos/larvae. However, our research has shown that when examining the marker produced by our team, there is an obvious need to administer it into the yolk or intravenously into the larva.

## 4. Materials and Methods

### 4.1. GCQD Synthesis

Carbon quantum nanodots were obtained by hydrothermal synthesis. The precursor solution (p.a. Warchem, Warsaw, Poland)) was prepared by dissolving 0.4375 g of sucrose and 0.0057 g of gadolinium (III) chloride 6 hydrate (p.a. Onyxmet, Olsztyn, Poland) in 25 cm^3^ of demineralized water (Polwater DL3N-150, Krakow, Poland)). The amounts of substrates were selected so that the ratio of carbon molecules from sucrose to gadolinium ions in the solution was approximately 100:1. It was assumed that sucrose was completely converted into carbon nanodots and a single carbon nanodot consists of 100 carbon atoms, which gives one gadolinium atom per nanodot. The precursor solution was also prepared for other carbon-to-gadolinium ratios (see Table 6). The aim was to obtain carbon nanodots doped to various degrees with gadolinium atoms. The prepared solutions were subjected to hydrothermal synthesis in a pressurized microwave reactor Ertec Magnum II (Ertec, Wrocław, Poland). The reaction vessel was made of PTFE. The maximum temperature during synthesis was 200 °C, at a maximum pressure of 45 bar. The synthesis time was 1 h. The solutions after synthesis were amber in color and clear. They had a noticeable, caramel smell. The obtained colloids were stored in sealed containers at a temperature of 10 °C.

### 4.2. Dialysis of CQDs and Gadolinium-Doped CQDs

Selected colloids were also made in a dialyzed version. Immediately after the reaction, the solution was purified by dialysis for 24 h. Dialysis used tubes with a regenerated cellulose membrane (Medicell International Ltd., London, UK, MWCO 12-14000 Daltons) with a wall thickness of 0.03 mm, a diameter of 28.6 mm, and a length of about 30 cm. This process allows for the removal of other hydrothermal synthesis products from the post-reaction mixture and forces the desorption of gadolinium ions bound to the surface of carbon nanoparticles. This procedure is significant for these studies.

A one-step synthesis process of Gd-modified carbon quantum dots (CQDs) leads to the formation of a structure where some of the Gd atoms are located on the surface while others are embedded within the CQDs. It is important to note that for CQDs, the proportion of surface atoms is high, making it relatively easy to remove surface dopants through dialysis. Consequently, both dialyzed and non-dialyzed CQDs were used in the studies, and their toxicity and properties were compared.

The synthesis process was efficient and streamlined, allowing for the incorporation of gadolinium atoms into the carbon quantum dot matrix. This method ensured that Gd atoms were distributed both on the exterior and within the interior of the CQDs, which can significantly influence the properties of the resulting nanomaterial. However, it should be noted that in the case of nanomaterials, the influence of surface atoms is meaningful. Calculations show that for a 2-nanometer-diameter gold nanoparticle, the ratio of surface atoms to interior atoms is 1:1. Therefore, the surface composition of the synthesized nanodots must be considered.

**Figure 12:**
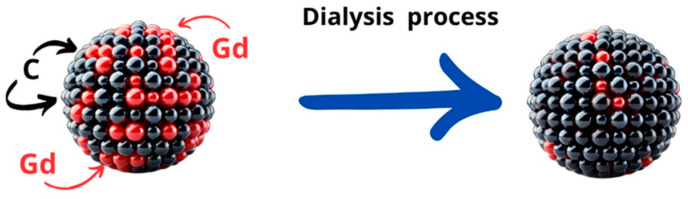
Visualization of the gadolinium ion removal from the CQD surface during dialysis process.

Given the high surface area of CQDs, the surface-bound Gd atoms can be selectively removed through dialysis (see Figure 12), which is a purification technique used to separate small molecules and ions from larger molecular complexes.

In comparative studies, the dialyzed CQDs—which had most of the surface Gd atoms removed—were analyzed alongside the non-dialyzed CQDs to assess their relative toxicity. This comparison is crucial for understanding the impact of surface versus internal Gd atoms on the overall biocompatibility and safety of the CQDs. The findings from these studies provide valuable insights into the design and application of Gd-modified CQDs in various fields, including medical imaging and drug delivery, where minimizing toxicity is of paramount importance.

Collides were then tested for gadolinium content in solutions before and after dialysis. The concentration of gadolinium ions was measured using an emission spectrometer with microwave-induced plasma Agilent MP-AES 4200 (Agilent, Santa Clara, CA, USA).

### 4.3. Characterization of Colloids

UV–Vis spectra were obtained using a Shimadzu model U-2501 PC spectrophotometer (Kyoto, Japan) within the wavelength range of 190–900 nm. Baseline correction was conducted with a reference cuvette containing deionized water. Spectra were measured with a 1-nanometer slit width using standard 10 mm or 2 mm quartz cuvettes (Hellma Analytics, Santa Clara, CA, USA).

High-resolution transmission electron microscopy (HR-TEM) was carried out using an FEI TECNAI TF 20 X-TWIN instrument (FEI Company, Hillsboro, OR, USA). A drop of freshly prepared colloidal suspension was placed onto a copper grid pre-coated with a 20–30-nanometer amorphous carbon film. The samples were allowed to dry at room temperature. Before HR-TEM analysis, all samples were subjected to dialysis to remove small molecules that could potentially cause carbon contamination.

The hydrodynamic diameter, size distribution, and zeta potential of the CQDs were measured using a Zetasizer Nano ZS (Malvern Instruments, Worcestershire, UK). The measurements utilized a standard clear polycarbonate cell with gold electrodes. Fluorescence spectra were recorded using a Horiba FluoroMax 4 spectrometer (Horiba, Kyoto, Japan) with a 1 cm 4-wall transparent quartz cuvette.

Selected colloids were analyzed to determine the quantum yield (Jasco FP-8500 Spectrofluorometer, Kyoto, Japan). The fluorescence lifetime was determined using a Horiba DeltaFlex instrument (Horiba, Kyoto, Japan) with the time-correlated single-photon counting (TCSPC) method. For the measurements, a DeltaDiode pulsed laser (λ_em_ = 371 nm) served as the excitation light source, and a quartz cuvette with a 1 cm optical path length was used.

### 4.4. Magnetic Properties Determination

The analysis of magnetic properties was carried out using the author’s test stand [39]. The methodology consisted of measuring the force with which the sample interacted with the magnetic field. The result was recorded in the form of an apparent mass change. A tested solution was placed in a quartz cuvette. Then, the sample was placed on an analytical balance (Ohaus PA214CM/1, Parsippany, NJ, USA) on an element separating the balance from the magnetic field. A set of neodymium magnets with a field strength of 0.5387 T, attached to a stepper motor, was brought closer to the prepared sample. The force with which the sample interacted with the magnetic field was recorded by the balance in the form of an apparent change in mass as a function of time of measurement or the distance of the magnet from the sample surface. The maximum recorded value of the apparent mass change corresponds to the relative magnetic susceptibility of the sample.

### 4.5. Chemicals

MS-222 was purchased from Sigma-Aldrich (Darmstadt, Germany). Gadovist (gadobutrol, Bayer Pharma, Berlin, Germany) was used as a control contrast agent with gadolinium.

### 4.6. Experimental Animal

The zebrafish embryos were obtained by the natural spawning of adult zebrafish (line Casper), which were housed in a continuous recirculating closed-system aquarium with a light/dark cycle of 14/10 h at 28 °C. Breeding was carried out in Zebrafish Core Facility of Jagiellonian University in Krakow, Institute of Zoology and Biomedical Research, Department of Evolutionary Immunology. The Jagiellonian University Zebrafish Core Facility is a licensed breeding and research facility (District Veterinary Inspectorate in Krakow registry and Ministry of Science and Higher Education, record numbers 022 and 0057). All experiments were conducted in accordance with the European Community Council Directive 2010/63/EU for the care and use of laboratory animals of 22 September 2010 (Chapter 1, Article 1 no.3) and National Journal of Law act of 15 January 2015 for the *Protection of Animals Used for Scientific or Educational Purposes* (Chapter 1, Article 2 no.1). All methods involving zebrafish embryos/larvae were in compliance with ARRIVE guidelines. All animals were handled in strict accordance with good animal practice as defined by the relevant national and/or local animal welfare bodies.

### 4.7. Embryos Collection

The embryos were collected and washed using standard zebrafish E3 culture medium (5 mmol/L NaCl, 0.33 mmol/L CaCl_2_, 0.33 mmol/L MgSO_4_·7H_2_O, and 0.17 mmol/L KCl). At 4 h post-fertilization (hpf), the embryos were examined under a dissecting light microscope (Stemi 305, ZEISS, Jena, Germany) and the specimens that had developed normally were selected for the further experiments according to the description of Kimmel et al. [40].

### 4.8. Zebrafish Toxicity Test

The acute hazard potential of GCQDs was determined based on the FET guideline [41], which provides a framework for the test. All tests were repeated two times. The total number of embryos (N) used was 630, 36–54 for each group.

The zebrafish embryos were exposed to the GCQDs for 4–76 hpf. They were kept in 24-well plates with three embryos per well containing 1 mL of the solution. The plates were covered with plastic lids to prevent evaporation. The GCQDs solutions were renewed every 24 h. Mortality, hatching, and morphology were assessed and recorded using a CCD digital camera (DP70, Olympus, Tokyo, Japan) mounted on a microscope (Stemi 305, ZEISS, Oberkochen, Germany) every 24 h from 4 hpf to 76 hpf.

Incubation at a constant temperature (28 ± 1 °C) employed an incubator (VWR with air circulation and photoperiod). Percentage of hatch (% hatch success) was defined as follows: (the number of larvae/initial number of embryos) × 100. The toxicity was assessed every 24 h for 76 h hpf: the malformation, heartbeats, spontaneous activity, and mortality of the zebrafish were observed using a stereoscopic dissecting microscope (Stemi 305, ZEISS, Oberkochen, Germany). The heart rate (72 h after exposition, 72 he) and spontaneous activity (48 and 72 he) were measured using a two-point scale (zero-one). Each embryo or larva was observed for 60 s. For rhythm disturbances, such as tachycardia, and for the lack of movement during this period, zero points were awarded. One point was awarded for a steady heart rhythm or spontaneous movements. Death was defined by cessation of heartbeat or coagulation of the embryos. The residues such as dead embryos were promptly removed.

**Figure 13:**
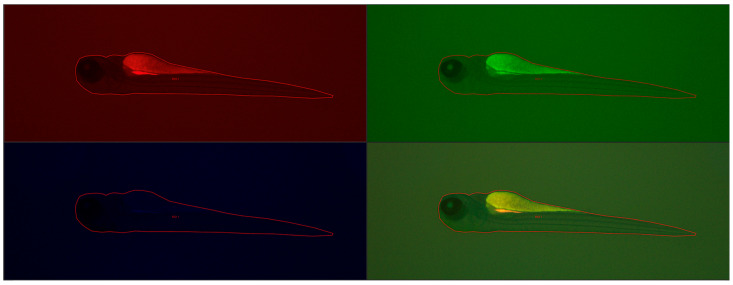
Example images from the analysis of the fluorescence signal using at blue, green and red wavelengths and merged (bottom right) in 4 dpf fish larvae. ROI: region of interest.

Next, the larvae (72 he) were washed three times with the E3 culture medium, anesthetized using 0.1 mg/mL MS-222 (Sigma-Aldrich, Darmstadt, Germany), and then they were visualized with a Leica DMI8 fluorescence inverted microscope (Leica Microsystems GmbH, Wetzlar, Germany). The images were captured with a digital Leica DFC450 CCD camera and Leica DMI8-CS inverted fluorescence microscope (Leica Microsystems GmbH, Wetzlar, Germany). All images were captured with the same settings (condenser: BF; exposure time: 324.951 ms; gain: 10; format 1920 × 1440 pixels; objective: 2.5). No correction was applied to the images. Assays were carried out to evaluate any changes compared to control larvae. Larval fluorescence intensity was calculated using Fiji software 2.15.1 (ImageJ2, GitHub, San Francisco, CA, USA), an open-source platform for biological-image analysis [42]. We calculated the fluorescence signal intensity by marking regions of interest (ROI) on the body surface of each larva fish and comparing it to the background signal (n = 5). Figure 13 shows example images from the analysis of the fluorescence signal.

### 4.9. Permeability through Chorions, Absorption into Embryos/Larvae, Content of Gadolinium in the Body

#### 4.9.1. Incubation and Collection of Embryo/Larvae

A total number of 300 *Danio rerio* embryos were used to test the content of gadolinium in the body after incubation with the tested solutions. Ten embryos were placed in each well of 6-well plates and then incubated for 24 h, 48 h, and 72 h with 2 mL of the tested solutions. The tested solutions were renewed every 24 h. Incubation was carried out in an incubator under constant conditions, T = 28 °C. The embryos or larvae were then collected in a plastic tube and euthanized by incubation with 1 mL of MS-222 at a concentration of 10 mg/mL at E3. After approximately 15 min, the embryos or larvae were washed three times with E3 (1 mL for 5 min). The buffer was then replaced with 3.8% formaldehyde in methanol and left for 24 h in the refrigerator. Next, larvae were successively washed twice in E3 buffer.

#### 4.9.2. Determination of Gadolinium Content in Zebrafish

The preparation in the amount of about 10 larvae per measurement series was filtered to separate the liquid phase. Then, the material together with the filters were placed in a ceramic crucible and placed in a furnace to remove volatile substances. The firing process was carried out in a chamber furnace in two stages, first raising the temperature to 200 for 3 h and holding for 2 h. Then, the temperature was raised to 700 for 3 h and held at this temperature for 6 h. In this way, graphitization of carbon was avoided, which allowed for its complete removal. Then, the samples were mineralized using a mixture of acids: 6 M nitric acid (V) and 2 M hydrochloric acid in the amount of 2.5 cm^3^ per sample. The solutions were then subjected to MP-AES spectrometric analysis.

### 4.10. Statistical Analysis

Statistical calculations were performed using GraphPad Prism 7 software (GraphPad Software, La Jolla, CA, USA). The results were grouped from three wells (9 embryos, larvae), resulting in n = 4–6 for each group. Results were expressed as mean ± SD and median. The non-parametric Kruskal–Wallis test and Dunn’s post hoc test were used for statistical calculations. Differences were considered at *p* < 0.05 (*).

## 5. Conclusions

Our research demonstrates the successful synthesis of gadolinium-doped carbon quantum dots (GCQDs) with both fluorescent and magnetic properties. Although the magnetic properties of the GCQDs were not as pronounced as traditional gadolinium-based agents like gadobutrol, our results highlight the potential for developing dual-contrast agents. This work opens new directions and possibilities for integrating carbon quantum dots (CQDs) with magnetic ions, aiming for dual functionality in diagnostic imaging.

The feasibility of incorporating gadolinium ions into the CQD matrix was confirmed, creating materials with both optical and magnetic properties. However, optimizing the synthesis process to enhance the incorporation efficiency of dopants, such as gadolinium or holmium, remains a challenge. Current limitations in achieving higher gadolinium content within CQDs underscore the need for further refinement in synthesis techniques.

Improving the purification and concentration processes for GCQDs is essential. Dialysis and other purification methods must be optimized to ensure that the resultant nanodots are free from unwanted byproducts and maintain consistent gadolinium content.

Toxicological assessments using zebrafish embryos provided promising results, confirming the low toxicity of gadolinium-doped CQDs. This finding is a milestone, indicating that these novel nanomaterials can be safely utilized in biological systems. The zebrafish model proved to be a reliable and convenient system for evaluating the potential nanotoxicity of GCQDs, showing comparable survival rates to control groups with minimal malformations.

In summary, while our results are not yet groundbreaking, they provide a solid foundation for further research. The dual-contrast potential of GCQDs is evident, and future work should focus on optimizing synthesis methods and enhancing the incorporation of magnetic dopants. Additionally, refining purification and concentration techniques will be crucial for producing high-quality GCQDs. Our study confirms the low toxicity of gadolinium-doped CQDs, marking a significant step toward their application in medical imaging. Continued research in this area holds promise for developing more effective and safer dual-contrast agents for diagnostic purposes.

## Figures and Tables

**Table 1:** Values of the strength of interaction of solutions with a magnetic field.

Sample Name *	Gadolinium Concentration [mol/dm^3^]	Apparent Δm [g]
CQD	0	0.0001
GCQD_1	0.0005	0.0004
GCQD_2	0.001	0.0012
GCQD_3	0.0015	0.0006
GCQD_4	0.002	0.001
GCQD_5	0.0025	0.0008
GCQD_10	0.005	−0.0064
GdCl_3_	0.00625	−0.0065
gadobutrol	1	−0.4614

* numbers in the sample name denote Carbon:Gadolinium atomic ratio: GCQD_1—100:1; GCQD_2—100:2; GCQD_3—100:3; GCQD_4—100:5; GCQD_5—100:5; GCQD_10—100:10.

**Table 2:** Gadolinium loss after dialysis depending on its initial concentration in CQDs.

Sample Name *	Gadolinium Loss after Dialysis [%]	Carbon:Gadolinium Atomic Ratio after Dialysis
GCQD_2	62.7	100:0.75
GCQD_10	14.7	100:8.5

* GCQD_2—Carbon:Gadolinium atomic ratio 100:2; GCQD_10—Carbon:Gadolinium atomic ratio 100:10.

**Table 3:** Gadolinium content in embryos after 24 h incubation with tested solution.

Sample Name *	Gadolinium Content per Sample [μg]	Gadolinium Content per Embryo [μg]	Gadolinium Content per Embryo Dry Mass [%]
control sample	0	0	0
CQD	0	0	0
GCQD_2	0	0	0
GCQD_10	0.125	0.0125	0.0200
GCQD/5_2	0.025	0.0028	0.0036
GCQD/5_10	0.075	0.0075	0.0120
GCQD_2_dial	0.100	0.0100	0.0160
GCQD_10_dial	0.125	0.0125	0.0200
GdCl_3_	0.050	0.0063	0.0064
gadobutrol	0	0	0

* Carbon:Gadolinium atomic ratio: CQD—100:0; GCQD_2—100:2; GCQD_10—100:10; GCQD/5_2—20:2; GCQD/5_10—20:10; GCQD_2_dial—100:2 + dialysis; GCQD_10_dial—100:10 + dialysis.

**Table 4:** Gadolinium content in embryos/larvae after 48 h incubation with tested solution.

Sample Name *	Gadolinium Content per Sample [μg]	Gadolinium Content per Embryo [μg]	Gadolinium Content per Embryo Dry Mass [%]
control sample	0	0	0
CQD	0	0	0
GCQD_2	0	0	0
GCQD_10	0	0	0
GCQD/5_2	0.075	0.0075	0.012
GCQD/5_10	0.175	0.0175	0.028
GCQD_2_dial	0.075	0.0075	0.012
GCQD_10_dial	0.075	0.0083	0.011
GdCl_3_	0.2	0.0200	0.032
gadobutrol	0	0	0

* Carbon:Gadolinium atomic ratio: CQD—100:0; GCQD_2—100:2; GCQD_10—100:10; GCQD/5_2—20:2; GCQD/5_10—20:10; GCQD_2_dial—100:2 + dialysis; GCQD_10_dial—100:10 + dialysis.

**Table 5:** Gadolinium content in larvae after 72 h incubation with tested solution.

Sample Name *	Gadolinium Content per Sample [μg]	Gadolinium Content per Embryo [μg]	Gadolinium Content per Embryo Dry Mass [%]
control sample	0	0	0
CQD	0	0	0
GCQD_2	0	0	0
GCQD_10	0	0	0
GCQD/5_2	0	0	0
GCQD/5_10	0	0	0
GCQD_2_dial	0	0	0
GCQD_10_dial	0	0	0
GdCl_3_	0	0	0
gadobutrol	0	0	0

* Carbon:Gadolinium atomic ratio: CQD—100:0; GCQD_2—100:2; GCQD_10—100:10; GCQD/5_2—20:2; GCQD/5_10—20:10; GCQD_2_dial—100:2 + dialysis; GCQD_10—100:10_dial + dialysis.

**Table 6:** Atomic fraction of elements in synthesized colloids.

Sample Name	Carbon:Gadolinium Atomic Ratio	Gadolinium Concentration
CQD	100:0	0
GCQD_1	100:1	0.0005
GCQD_2	100:2	0.001
GCQD_3	100:3	0.0015
GCQD_4	100:4	0.002
GCQD_5	100:5	0.0025
GCQD_10	100:10	0.005
GCQD/5_2	20:2	0.001
GCQD/5_10	20:10	0.005

## Data Availability

All data are available on request from the corresponding author.
